# Short-Term and Long-Term Effects of Bariatric Surgery on Diabetic Retinopathy: A Systematic Review

**DOI:** 10.3390/medicina61010157

**Published:** 2025-01-18

**Authors:** Erika Butkutė, Monika Zieniūtė, Agnė Morkūnaitė, Vilma Jūratė Balčiūnienė

**Affiliations:** 1 Medical Academy, Lithuanian University of Health Sciences, Eiveniu 2, LT-50161 Kaunas, Lithuania; 2 Department of Ophthalmology, Medical Academy, Hospital of Lithuanian University of Health Sciences Kaunas Clinics, LT-50161 Kaunas, Lithuania

**Keywords:** diabetic retinopathy, type 2 diabetes mellitus, bariatric surgery, metabolic surgery

## Abstract

*Background and Objectives*: Diabetic retinopathy (DR) is a common diabetes complication and a leading cause of blindness. Although bariatric surgery (BS) is well studied for diabetes management, its effects on DR onset and progression, particularly long-term outcomes, remain underexplored. This review seeks to evaluate the short- and long-term retinal outcomes of BS in diabetic patients. *Materials and Methods*: A systematic search of PubMed, Web of Science, and Cochrane Library was conducted following PRISMA 2020 guidelines, including the use of the PRISMA checklist and flow diagram. The search included English-language articles (2014–2024) using keywords like “diabetic retinopathy” and “bariatric surgery”. The review excluded studies published in languages other than English, as well as reviews, case reports, and those lacking adequate data or relevance. The risk of bias was determined by using the Downs and Black checklist. A systematic review of the literature was conducted, and the results were organized and displayed in tables to summarize and compare the main findings. *Results*: After screening 158 articles, 13 studies involving 17,903 patients were selected, focusing on the effects of BS on DR progression and regression. Short-term data suggest that BS may stabilize or improve DR but poses risks of worsening in advanced cases, requiring careful monitoring. However, long-term findings are inconsistent, with evidence of both DR regression and progression. These variations highlight the need for further research. *Discussion*: BS generally improves DR progression, but its effect depends on preoperative severity and glycemic control. Further studies should explore additional risk factors to better predict DR outcomes post-surgery. *Conclusions*: BS improves weight management, blood pressure, and diabetes control, potentially benefiting DR. However, the long-term effects remain uncertain due to conflicting findings. Rapid glycemic changes after surgery may pose risks of DR progression. Large-scale, longitudinal studies are needed to clarify the role of BS in DR progression and regression.

## 1. Introduction

The worldwide prevalence of type 2 diabetes has been steadily rising and is often referred to as a pandemic. In 2017, the condition affected over 462 million people and was projected to become the ninth leading cause of death [1]. Diabetic retinopathy (DR) is the most common diabetes-related complication and a major cause of blindness [2]. Various risk factors have been shown to play a key role in the development of DR, including modifiable ones such as high blood sugar, high blood pressure, elevated lipid levels, and obesity, as well as non-modifiable factors like the duration of diabetes and pregnancy [3]. Bariatric surgery (BS) is a well-recognized treatment for individuals with diabetes and severe obesity. In addition to promoting significant weight loss, it profoundly alters the patient’s metabolic profile, improving lipid levels and insulin sensitivity, and may even result in diabetes remission in some cases after surgery [4,5]. The cost of managing diabetes is estimated to be 20 times higher for patients with four or more diabetes-related complications compared to those with no complications, making it an additional burden in managing the disease effectively [6,7].

Several studies have explored the impact of BS on patients with diabetes, but less focus has been placed on its effect on the onset and progression of microvascular complications, particularly DR [8,9,10,11]. Given the rising prevalence of both obesity and diabetes, along with the increasing adoption of BS, understanding its impact on DR is essential. However, existing studies have yielded conflicting results regarding both the short-term and long-term effects of BS on DR outcomes. Therefore, a comprehensive review of current evidence is necessary to identify whether BS can offer sustained benefits or pose risks for DR [12,13].

The purpose of this review is to explore scientific articles published since 2014, analyzing both the short-term and long-term effects of BS on the course of DR in patients with diabetes.

## 2. Materials and Methods

This systematic review adhered to the guidelines established by the Preferred Reporting Items for Systematic Reviews (PRISMA 2020), including the use of the PRISMA checklist and flow diagram [14]. The following PICO strategy was established:

The participants (P): patients with type 2 diabetes mellitus (T2DM);
The intervention (I): bariatric surgery (BS) (all types of procedures);
The comparison (C): short-term (<3 years) and long-term (≥3 years) outcomes of BS in patients with diabetic retinopathy (DR);
The outcomes (O): % of new incidence of any DR in patients with no retinopathy at baseline; % of worsening and % of improvement of DR in patients treated by BS during the follow-up period; and % of no change in DR status.

### 2.1. Search Strategy

A systematic search was conducted using the PubMed, Web of Science, and Cochrane Library databases. The search strategy employed the following keywords: “diabetic retinopathy”, “type 2 diabetes mellitus”, and “bariatric surgery” or “metabolic surgery”. The search was restricted to English-language articles published within the last 10 years (2014–2024). No limitations were placed on the country of publication or publication status. Two authors independently reviewed the titles and abstracts to assess eligibility based on the inclusion and exclusion criteria. Two authors independently extracted data on patient demographics, DR status, and BS outcomes using a standardized data extraction form. Full-text articles were then independently reviewed and evaluated following this initial screening. In the event of a disagreement, a third author was consulted to reach a final decision. The data and analysis code used in this systematic review can be obtained from the corresponding author upon request. This review was not pre-registered in any publicly available registry. While a protocol was developed internally, it is not publicly accessible.

### 2.2. Selection Criteria

Full-text articles were obtained for all potentially relevant studies. The inclusion criteria for this review were studies published in English, focusing on observational, multicenter, comparative, and cohort studies, as well as research-supported studies and clinical trials, with only freely accessible publications considered. The exclusion criteria were as follows: non-English studies, letters, editorials, meeting abstracts, reviews, case series, and case reports. Additionally, studies that failed to address the research questions or lacked sufficient data regarding pre- and post-DR status were not included.

### 2.3. Risk of Bias

Two independent authors evaluated the quality of the included studies. The risk of bias was determined using the Downs and Black checklist [15]. This checklist contains 27 questions, each scored as 0 or 1, with 0 indicating the criterion is not met and 1 indicating that it is met. Studies meeting fewer than 14 criteria were considered of poor quality, those meeting 15–19 criteria were classified as fair, 20–25 as good, and more than 25 as excellent. The included studies scored between 15 and 21 points for quality. The risk of bias was assessed independently by two authors, with a third author consulted to resolve any disagreements or discrepancies in the evaluations. Detailed scoring and individual study assessments are presented in Table 1.

### 2.4. Data Synthesis

The included studies were synthesized qualitatively, comparing results based on new-onset DR, DR worsening, DR improvement, and no change in DR status across short-term and long-term follow-up periods. Due to the significant heterogeneity in study designs, populations, intervention types, and outcome measurements, a meta-analysis was not feasible. The results were summarized descriptively to capture common trends and patterns across the studies. This approach ensures that the differences in methodologies are acknowledged and facilitates their interpretation within the context of the reviewed studies.

## 3. Results

### 3.1. Research Identification and Selection

The current systematic review included a total of 13 studies conducted between 2016 and 2023 across six countries, as presented in Table 2.

**Figure 1:**
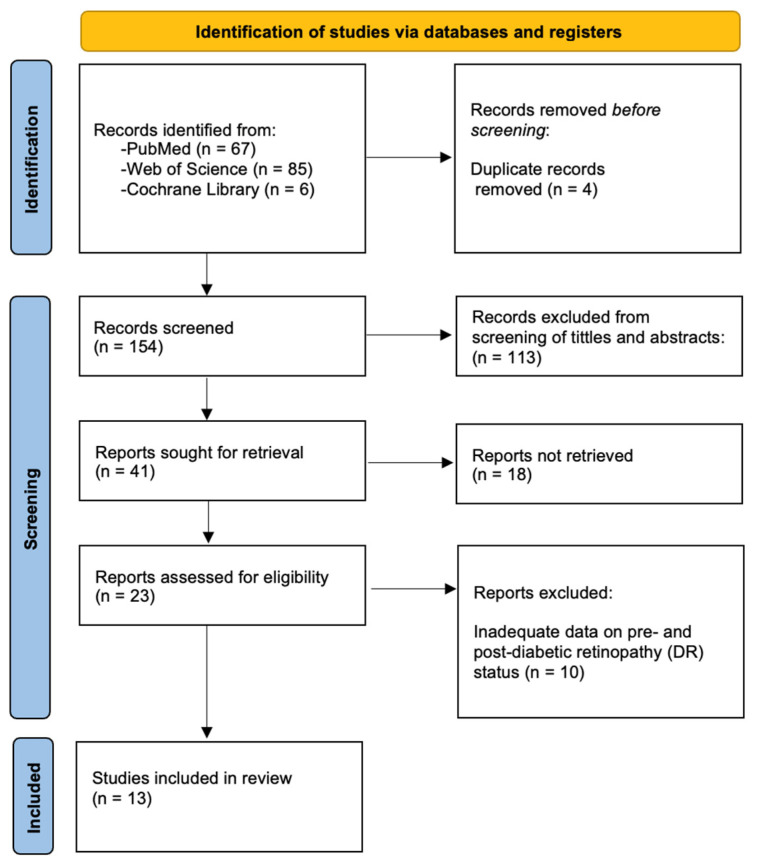
Diagram of the search process.

The research selection process adhered to the PRISMA (Preferred Reporting Items for Systematic Reviews) recommendations. A flow chart is provided in Figure 1. The process of research identification began with a thorough search on three electronic databases, including PubMed, Web of Science, and Cochrane Library. This search was carried out using a combination of keywords, including “diabetic retinopathy”, “type 2 diabetes mellitus”, and “bariatric surgery” or “metabolic surgery”. The search resulted in the identification of a total of 158 papers: 67 studies from PubMed, 85 studies from Web of Science, and 6 studies from Cochrane Library. After eliminating duplicates, 154 articles remained. After screening their titles and abstracts, 113 were discarded for not meeting the inclusion and exclusion criteria set by the authors. Following a thorough review of the full texts, 18 studies were excluded due to irrelevance to the study topic, unavailability of full texts, or not answering the research question. Following this step, a total of 23 articles remained for comprehensive reading. Finally, after a complete review, 13 articles, involving 17,903 patients, were selected for synthesis. The studies varied in sample size, ranging from 21 to 5321 participants. The types of BS investigated included gastric bypass surgery, vertical sleeve gastrectomy, Roux-en-Y gastric bypass, laparoscopic sleeve gastrectomy, and laparoscopic adjustable gastric banding. The follow-up duration across the studies varied from 1 month to 6 years.

We assessed the quality of the included studies using the Newcastle–Ottawa Scale (NOS) [29]. This scale evaluates studies based on three main criteria: selection, comparability, and outcome assessment. The scores of the studies, ranging from seven to nine, indicating that the majority of studies were of moderate-to-high quality, are provided in Table 2.

### 3.2. Diabetic Retinopathy

The most common microvascular complication of type 2 diabetes mellitus (T2DM) globally is diabetic retinopathy (DR), which is also the main cause of visual impairment in adults aged 20 to 74 [8,30,31]. Even though effective treatments exist, the incidence of blindness caused by DR is rising. The worsening of vision due to DR will impose a significant burden on both individuals and the economy, especially in middle-income nations [32]. In the beginning, DR is usually asymptomatic, with only a few symptoms appearing before vision impairment develops; therefore, regular comprehensive eye exams are essential for both patients with type 1 and type 2 diabetes [33,34]. DR is differentiated into non-proliferative (NPDR) and proliferative types (PDR), based on whether or not abnormal new blood vessels develop in the retina. NPDR is classified as mild, moderate, and severe, while PDR is classified as early or high-risk [34]. NPDR is the early phase of DR. Advanced stages of PDR can be complicated by neovascularization—the process of abnormal blood vessel growth in the retina—which can result in hemorrhage, retinal detachment, and, consequently, vision loss. Another serious complication of DR is diabetic macular edema, which is the leading cause of vision loss in patients with DR [35]. Patients with severe NPDR and early PDR can undergo laser therapy or anti-VEGF treatment, while those with high-risk PDR may require surgical removal of the vitreous. Regardless of the stage of DR, maintaining blood sugar, blood pressure, and cholesterol levels is important to prevent progression. Effective metabolic control in diabetic patients is crucial for successful DR treatment [36].

### 3.3. Bariatric Surgery

Bariatric surgery (BS) is widely recognized as the most effective long-term solution for treating obesity and can also lead to remission of type 2 diabetes (T2D) [37]. According to the guidelines of the National Institute for Health and Care Excellence (NICE), BS is recommended for individuals with a BMI over 40, or between 35 and 40 if they have a serious health condition like T2D or hypertension that could be improved through weight loss [19]. The most performed bariatric procedures include Roux-en-Y gastric bypass (RYGB), laparoscopic sleeve gastrectomy (SG), and laparoscopic adjustable gastric banding (LAGB) [13]. RYGB reduces the stomach’s capacity by forming a small gastric pouch, which is then connected to the mid-jejunum, leading to reduced food absorption [38]. SG involves removing the larger part of the stomach, leaving behind a narrow, tube-shaped stomach, which limits food intake [39]. LAGB places a band around the upper part of the stomach, and this band is connected to a port under the skin, allowing for adjustments to tighten the band and further limit stomach capacity [40]. Each of these procedures comes with its own set of advantages and potential risks. Each procedure enhances insulin sensitivity, reduces insulin resistance, and can decrease blood sugar levels [41,42]. Some patients experience remission of diabetes shortly after surgery, even before significant weight loss occurs, due to hormonal changes that improve glucose metabolism [43,44,45]. As a result, many patients can reduce or eliminate their need for diabetes medications after BS [46]. Among various obesity treatments, BS is associated with the most significant and lasting weight loss, as well as positive effects on blood sugar control and other cardiovascular risk factors [23]. The overall effect of BS on retinal microvasculature is still not well understood, and research findings on its potential impact on DR are mixed [22,47,48,49].

### 3.4. Bariatric Surgery: Short-Term Outcomes on Diabetic Retinopathy

Bariatric surgery (BS) results in unpredictable changes in diabetic retinopathy (DR) [50]. Considering the known impact on DR from rapidly correcting blood sugar levels with medications, there is growing concern about the rapid and significant decreases in blood glucose levels that often follow BS [8,51]. Several studies [16,17,18,19,20,21,22] have been carried out to investigate the short-term impact of BS on retinal outcomes in patients with type 2 diabetes and obesity.

In 2016, a prospective observational study was conducted by Brynskov et al. [16]. This study involved 56 patients who underwent either RYGB or vertical sleeve gastrectomy (VSG). Over a year, six patients experienced a worsening of DR, but this condition only persisted in three individuals at the 12-month mark. While 17 of the 24 patients with preoperative DR maintained their condition, three experienced worsening, and four improved. For those without preoperative DR, 1 of 30 participants experienced a temporary worsening at 6 months, but at 12 months, all 30 participants remained unchanged from their baseline condition. Based on these findings, the study concluded that retinopathy remained clinically stable during the first year following BS [16]. Morén et al. [17] estimated the results related to the occurrence and severity of DR before and after BS and examined possible risk factors for DR worsening. Twelve patients (16%) developed new-onset (mild) DR. In seven patients (16%) with pre-existing DR, worsening of DR was observed. Among patients with pre-existing DR, 8 of 44 (18%) improved at least one step on the DR grading scale. Twelve months after gastric bypass surgery (GBP), diabetic treatment was discontinued in 77 of 117 patients (66%). Despite the rapid improvement in metabolic control after surgery, most patients did not experience a change in DR occurrence. Furthermore, the study found no correlation between preoperative HbA1c, BMI, BMI reduction, or HbA1c reduction and DR progression, and did not identify specific risk factors for worsening of DR post GBP [17]. The retrospective cohort study by Wenhuan Feng et al. [18] included 40 patients who underwent RYGB and 36 patients who were administered conventional medication (CM). In this study, pre- and post-DR status were analyzed. Before the surgery, 15% of patients in the BS group were diagnosed with DR and this number decreased to 8% after one year. The medication group showed no change in DR status over the same period [18]. Ozkan Sever and colleagues [19] examined the changes experienced by patients with PDR after BS in a retrospective observational study. Both groups had statistically significant visual acuity (VA) losses at 6 months and 1 year. At the end of 1 year, the BS group required significantly fewer injections compared to the CG group, but they experienced a higher rate of complications, including intraocular hemorrhages, neovascular glaucoma, and retinal vein occlusions [19]. However, this is a small single study, so more research is needed to examine DR complications after BS. A cohort study was conducted by Åkerblom H. et al. [20] in 2021 in Sweden (n = 5321). In total, 188 patients (0.83%) in the GBP group and 317 patients (2.94%) in the control group developed new-onset DR. The incidence of developing DR was lower in the BS group. There was no significant difference in the development of sight-threatening diabetic macular edema between the GBP group and the control group (0.79% vs. 0.85%) [20]. Chandru S. et al. [21] examined microvascular changes associated with DR in obese Asian Indians with T2DM one year after metabolic surgery. In this study with 21 patients, 8 individuals (38%) presented with DR at baseline. Post-surgery follow-up revealed that the majority (87.5%) maintained stable disease. One individual achieved complete DR regression, and in one individual, there was a one-step regression from severe to moderate NPDR. However, one patient experienced a one-step progression from moderate to severe NPDR (12.5%) [21]. In the study by Anne S. Thykjaer [22] involving patients with type 2 diabetes, BS was associated with a lower incidence of DR worsening (worsening was defined) at 6 months (2.9% in surgery cases vs. 8.4% in controls). DR improvement (defined as 2-step improvement of DR) occurred in <45% of the patients in the case group compared to 19.7% in the control group [22]. The study suggests that BS may protect against the rapid progression of DR. All of these results are presented in Table 3.

Several studies have demonstrated that BS is associated with DR regression or stable DR in short-term follow-up [16,17,18,20,21,22]. Although there are limitations to short-term studies, these findings suggest a potential benefit of BS for managing DR.

### 3.5. Bariatric Surgery: Long-Term Effects on Diabetic Retinopathy

In a Danish nationwide cohort study, Anne S. Thykjær and colleagues examined the long-term effects of BS on DR [22]. Their findings revealed that the case population had stable glycaemic levels at baseline, along with a decrease in HbA1c levels both before and after surgery, which may account for the low rates of DR progression [22]. After 36 months, DR worsening and new cases of DR occurred in 5.2% of the patients in the case group compared to 7.9% in the control group [22]. The retrospective matched, controlled cohort study by Singh P. et al. demonstrated that BS was significantly linked to a reduction in new cases of diabetes-related microvascular complications when compared to individuals who did not undergo surgery [23]. An analysis by surgery type showed that all procedures—gastric banding (GB), sleeve gastrectomy (SG), Roux-en-Y gastric bypass (RYGB), and duodenal switch (DS)—had a positive effect on reducing the incidence of microvascular complications, including sight-threatening DR, in patients with type 2 diabetes and obesity [23]. After an average follow-up of 3.5 years, the non-surgical cohort (n = 96) had an incidence rate of 4.6%, while the surgical cohort (n = 34) had a lower incidence rate of 3.2% [23]. In a cross-sectional study by Madsen and colleagues, among 96 patients who underwent RYGB surgery, no worsening of retinopathy was observed over a 6-year follow-up period [24]. In contrast, a sample of 48 non-operated controls showed progression of retinopathy, as assessed using fundus photography [24]. In the cohort study by Miras et al., among a subgroup of 24 patients (29% of the total cohort) with available retinal images, retinal conditions were stable in 13 patients, worsened in 6, and improved in 5 patients five years after obesity surgery [25]. These patients had undergone various procedures, including RYGB, SG, and GB [25]. The authors discuss how complex hormonal and metabolic changes following BS might lead to rapid improvements in glycemic control even before significant weight loss occurs, potentially contributing to the early worsening of DR [25]. They also mention other factors that could influence DR progression, such as deficiencies in vitamins and micronutrients (A, D, B12, copper, and folate) due to malnutrition, as well as the discontinuation of oral antidiabetic medications that provide protection against retinal damage, like fenofibrate or angiotensin receptor blockers [13,25]. Richardson et al. analyzed 3-year changes in DR grade in a retrospective observational study of 32 morbidly obese patients (64 eyes) who underwent RYGB surgery. They found that despite overall benefits in vision, patients with pre-proliferative DR at baseline were at increased risk of developing sight-threatening DR [26]. In the retrospective observational study by Chen Y. et al., a notable progression in DR was observed. Among the 69 eligible patients, 24 (34.8%) developed new cases of DR, while 2 out of 33 patients (6.1%) experienced progression of existing retinopathy during a follow-up period of 4 years [27]. In a 2016 study by Amin and colleagues, the effects of BS on DR were analyzed in 152 obese patients with type 2 diabetes over a 3-year period following surgery [28]. Most patients underwent GB, while a smaller number received RYGB and SG [28]. Although the study did not provide specific numerical comparisons with a control group of patients receiving medical treatment, the authors concluded that, when compared to a matched group based on age, glycated hemoglobin levels, and follow-up duration, patients who underwent surgery experienced less progression to sight-threatening DR (STDR) than those who received routine care [28]. All of these results are presented in Table 4.

In conclusion, numerous studies indicate that BS can lead to regression of DR [22,23,24,25,28]. However, there are also studies reporting progression and new cases of DR after surgery, and these findings cannot be overlooked [26,27]. This disparity highlights that the long-term effects of BS on DR remain an area of ongoing research and investigation.

## 4. Discussion

The articles included in our review describe both onset and worsening, as well as stabilization and improvement, as outcomes for diabetic retinopathy (DR) following bariatric surgery (BS). Although the data on the prevailing tendency remain not entirely conclusive, certain patterns have emerged following recent studies, with the general consensus being that the effects of BS on DR are not unfavorable enough to render BS unsuitable for diabetic patients.

A diverse array of short-term DR outcomes has been reported. Several studies found that the majority of patients who underwent BS did not experience change in their DR status up to 1 year post BS [16,17,21,22]. Furthermore, Brynskov et al. even noted that at 6-month follow-up, a significant number of patients experienced improvement in their DR status [16]. In fact, at each follow-up visit, more patients showed improvement than experienced worsening. The authors attributed these findings to the early stage of DR and the good preoperative diabetic control within their cohort. Moreover, patients who experienced worsening had already been diagnosed with DR preoperatively, including proliferative DR (PDR). Similarly, Sever et al. observed that patients with PDR experienced short-term worsening in DR status [19]. Moreover, they proposed that the severity of preoperative DR may be a predictive factor for DR progression. Richardson et al. substantiated this hypothesis, reporting that in a cohort of patients who underwent RYGB, an initial worsening of DR was observed in some individuals, especially those with pre-existing proliferative DR [26]. They proposed rapid and marked improvements in blood glucose levels as the culprit for the early paradoxical worsening in DR status, as patients with advanced DR faced increased risks of progressing to sight-threatening DR (STDR) within the first year post surgery. Furthermore, Amin et al. emphasized that patients with pre-existing DR are more susceptible to STDR post BS, and even noted that DR onset and progression to STDR may still occur in some patients who had no DR before surgery [28]. Despite the mostly positive outcomes reported, the importance of close monitoring and cautious glycemic targets for diabetic patients in the early postoperative period is evident, especially for those with advanced DR.

In studies with longer follow-up periods, the prevailing outcomes for DR reported post BS were stabilization and improvement. Som authors agree that most patients who have undergone BS tend to experience better DR outcomes compared to non-operated individuals. Along with Amin et al., Madsen et al. found that overall, patients who received BS had slower DR progression when compared to non-operated controls [24,28]. Similarly, Singh et al. discovered that the incidence of sight-threatening DR was lower in the BS group compared to the control group, suggesting its positive effect on DR [23]. As demonstrated in short-term findings, Chen et al. observed that patients with preoperative DR were at increased risk of experiencing DR progression [27]. Furthermore, Amin et al. noted that although the BS group was less likely to progress to STDR compared to controls, patients with pre-existing DR remained at risk of developing STDR in the long term. In contrast, Amin et al., along with Chen et al., concluded that in the long term, BS does not eliminate the risk of developing DR or STDR, even in patients with no preoperative evidence of DR. Nevertheless, Richardson et al. have argued that, according to their study, the “net” improvement in DR outcomes in the long term outweighs the setback of short-term DR worsening. Given the multifaceted long-term impact of BS, predictive factors, other than preoperative DR status, should be considered.

Several possible predictive factors, related to DR status change post BS, have been noted by authors. Amongst them, the most widely discussed is glycemic control, along with preoperative HbA1c levels and their postoperative dynamic. Patients included in the studies by Brynskov et al. and Thykjær et al. had low pre-surgical HbA1c levels, which was theorized to have led to the low incidence of DR progression [16,22]. While Amin et al. noticed a trend where lower postoperative HbA1c levels were associated with lower rates of progression to STDR, the relationship was not statistically significant [28]. Morén et al., along with Miras et al., on the other hand, did not identify any predictive factors that could lead to an increased risk of DR post BS [17,25]. Moreover, some authors have argued that preoperative and postoperative weight changes are not associated with better DR outcomes [22,24,28]. Lastly, although not finding patient weight to be a statistically significant predictive factor, Chen et al. agreed that high preoperative HbA1c levels were associated with negative DR outcomes [27]. They also proposed additional criteria, such as younger age and male gender as predictive factors, suggesting that the post-BS change in DR status might be a multifactorial phenomenon (substantiated by the study by Sever et al. [19]) and that, beyond HbA1c levels, other yet-to-be-investigated predictive factors and their interplay with the already more established ones, like HbA1c, could serve as valuable areas for future research.

Diabetic retinopathy is a complicated disease involving both positive and negative mechanisms. These mechanisms include various physiological and biochemical pathways that can either contribute to the disease’s progression or complicate its development [35,52]. Understanding these processes is crucial for exploring potential interventions that can alter the course of the disease. 

Potential positive mechanisms include metabolic autoregulation; in the early stages of diabetic retinopathy (DR), the vascular dilation and changes in blood flow caused by hyperglycemia can be considered metabolic autoregulation, which aims to enhance retinal metabolism [35]. However, this compensatory mechanism is often insufficient to fully repair the damage caused by hyperglycemia [52]. In Müller cell regulation, according to the methanol–formaldehyde–acetic acid (MFA) hypothesis, activated Müller cells may release substances that dilate retinal blood vessels to improve circulation and supply oxygen to the neuroretina. This mechanism is part of the body’s response to hypoxia caused by the accumulation of acetic acid [52]. Potential negative mechanisms include damage induced by hyperglycemia; elevated blood glucose levels are a major factor in the development of diabetic retinopathy, initiating a cascade of events including microvascular damage, pericyte loss, and endothelial dysfunction, leading to increased vascular permeability and retinal edema [52]. One of the first signs of diabetic retinopathy is pericyte loss, which weakens the capillary walls and promotes the formation of microaneurysms. The absence of pericytes compromises the structural stability of capillaries, increasing their susceptibility to damage [35,52]. Hyperglycemia induces endothelial cell apoptosis, leading to disruption of the blood–retinal barrier (BRB). This breakdown increases vascular permeability, causing fluid accumulation in the retina and contributing to macular edema. Damage to endothelial cells and the loss of pericytes result in occluded capillaries, causing retinal ischemia and a lack of oxygen. This hypoxia triggers the release of vascular endothelial growth factor (VEGF), which stimulates the growth of abnormal blood vessels, a key feature of proliferative diabetic retinopathy [13,35,52,53]. Moreover, oxidative stress is a significant activator of inflammatory cascades, and both oxidative stress and inflammation promote retinal autophagy, resulting in dysfunction, necrosis, apoptosis, and cell death [54].

By improving glycemic control, bariatric surgery might slow or even reverse some of the damaging effects of hyperglycemia on retinal cells, vascular structures, and the blood–retinal barrier. On the other hand, understanding the negative mechanisms of DR, such as endothelial damage and the formation of microaneurysms, is important because these changes could still occur after bariatric surgery if metabolic control is not sustained or if other factors, such as oxidative stress, remain unchecked.

Several limitations should be acknowledged in this review: including studies published in languages other than English in future research could improve the comprehensiveness of the findings, offering a more global perspective on the effects of bariatric surgery on diabetic retinopathy. This would better represent the broad range of outcomes and interventions across different populations. In addition, the observed heterogeneity across the included studies limits the ability to draw conclusive and universally applicable findings. Future meta-analyses, incorporating studies with more consistent methodologies, could facilitate a more accurate and generalized assessment of the effects of bariatric surgery on diabetic retinopathy.

Overall, BS appears to offer favorable effects on DR progression in most diabetic patients. However, this benefit is influenced by additional factors like pre-existing DR severity and post-surgical glycemic control, which have the potential to modify the outcome post BS. Patients with higher-risk profiles—those with severe DR and/or high preoperative HbA1c levels—require tailored post-surgical monitoring and management. Further research should focus on exploring the link between patient-specific risk factors and DR progression following BS.

## 5. Conclusions

Numerous prospective and retrospective studies confirm that bariatric surgery (BS) is the most effective method for achieving significant weight loss, lowering blood pressure, regulating glycemia, and achieving long-term remission of diabetes. Additionally, BS is recommended to prevent the progression of both microvascular and macrovascular complications, including diabetic retinopathy (DR) [55]. However, the impact of BS on retinal health remains controversial. While some studies suggest that the procedure may have a positive long-term effect due to better blood pressure and glycemic control, others highlight the potential for rapid DR progression in the immediate postoperative period.

The progression of DR following BS may be influenced by various factors, particularly the sudden and significant reduction in blood glucose levels after surgery [56]. Although some reports do not show major differences in DR progression during long-term follow-up, it is important to emphasize that, for most patients, DR tends to remain stable after surgery.

Nevertheless, the reliability of the current data is limited. Most studies examining the effects of BS on DR in diabetes patients are retrospective, small in sample size, and methodologically inconsistent. While BS may help prevent new cases of DR, the available evidence is insufficient to conclusively determine whether the procedure influences the progression or regression of existing retinopathy. Further large-scale, methodologically robust studies are needed to draw definitive conclusions about the long-term impact of BS on DR.

## Figures and Tables

**Table 1:** Downs and Black checklist for quality assessment.

All CRITERIA	Brynskov T. et al. [16]	Morén et al. [17]	Wenhuan Feng et al. [18]	Ozkan Sever et al. [19]	Åkerblom H. et al. [20]	Chandru S. et al. [21]	Anne S. Thykjaer et al. [22]	Singh P. et al. [23]	Madsen et al. [24]	Miras, Alexander D. et al. [25]	Richardson et al. [26]	Chen Y. et al. [27]	Amin, Amin Mamoon et al. [28]
Q1. Hypothesis/aim/objective clearly described	1	1	1	1	1	1	1	1	1	1	1	1	1
Q2. Main outcomes in Introduction or Methods	1	1	1	1	1	1	1	1	1	1	1	1	1
Q3. Patient characteristics clearly described	1	1	1	1	1	1	1	1	1	1	1	1	1
Q4. Interventions of interest clearly described	1	1	1	1	1	1	1	1	1	1	1	1	1
Q5. Principal confounders clearly described	1	1	0	0	1	0	1	1	1	0	1	1	1
Q6. Main findings clearly described	1	1	1	1	1	1	1	1	1	1	1	1	1
Q7. Estimates of random variability provided for main outcomes	0	1	0	0	1	1	1	1	1	0	1	1	1
Q8. All adverse events of intervention reported	0	1	1	1	0	0	0	0	0	0	0	0	0
Q9. Characteristics of patients lost to follow-up described	0	1	0	0	1	0	0	1	0	0	1	0	0
Q10. Probability values reported for main outcomes	1	1	0	0	1	1	1	1	1	0	1	1	1
Q11. Subjects asked to participate were representative of source population	1	1	1	1	1	1	1	1	1	1	1	1	1
Q12. Subjects prepared to participate were representative of source population	1	1	1	1	1	1	1	1	1	1	1	1	1
Q13. Location and delivery of study treatment was representative of source population	1	0	1	1	1	1	1	1	1	1	1	1	1
Q14. Study participants blinded to treatment	0	0	0	0	0	0	0	0	0	0	0	0	0
Q15. Blinded outcome assessment	1	1	1	1	0	0	0	0	0	1	0	0	0
Q16. Any data dredging clearly described	1	1	1	1	0	0	1	1	1	1	1	1	1
Q17. Analyses adjust for differing lengths of follow-up	1	1	1	1	1	1	0	1	1	1	0	0	1
Q18. Appropriate statistical tests performed	1	1	1	1	1	1	1	1	1	1	1	1	1
Q19. Compliance with interventions was reliable	1	0	1	1	UTD	1	1	UTD	1	1	1	1	1
Q20. Outcome measures were reliable and valid	1	1	1	1	1	1	1	1	1	1	1	1	1
Q21. All participants recruited from the same source population	UTD	1	1	1	1	1	UTD	1	1	UTD	UTD	UTD	1
Q22. All participants recruited over the same time	UTD	1	1	1	1	1	UTD	1	1	UTD	UTD	UTD	1
Q23. Participants randomized to treatment(s)	UTD	UTD	1	0	0	0	UTD	0	0	UTD	UTD	UTD	0
Q24. Allocation of treatment concealed from investigators and participants	UTD	UTD	UTD	UTD	UTD	UTD	UTD	UTD	UTD	UTD	UTD	UTD	UTD
Q25. Adequate adjustment for confounding	0	UTD	1	1	1	1	0	1	1	1	0	0	1
Q26. Losses to follow-up considered	0	UTD	1	1	1	0	0	1	0	1	0	0	1
Q27. Sufficient power to detect treatment effect at significance level of 0.05	0	1	1	1	1	0	0	1	1	1	0	0	1
TOTAL	16	20	21	20	20	17	15	21	20	17	16	15	21
Quality	Fair	Good	Good	Good	Good	Fair	Fair	Good	Good	Fair	Fair	Fair	Good

UTD: unable to determine.

**Table 2:** Characteristics of included studies.

Study No.	Author(s)	Country	Year	Study Type	Sample Size (n)	Types of Bariatric Surgery	Follow-Up Duration	Quality Score (NOS)
1	Brynskov T. et al. [16]	Denmark	2016	Prospective observational clinical study	C: 56	RYGB; VSG	1, 3, 6, and 12 months	7
2	Morén et al. [17]	Sweden	2018	Multicenter cohort study	C: 117	GBP	1 year	8
3	Wenhuan Feng et al. [18]	China	2019	Retrospective cohort study	C: 76	RYGB	1 year	7
4	Ozkan Sever et al. [19]	Turkey	2020	Retrospective observational study	C: 21 (37 eyes) CG: 27 (37 eyes)	VSG	6 months and 1 year	9
5	Åkerblom H. et al. [20]	Sweden	2021	Cohort study	C: 5321 CG: 5321	GBP	1 year	8
6	Chandru S. et al. [21]	India	2022	Follow-up study	C: 21	Unspecified	1 year	8
7	Anne S. Thykjaer et al. [22]	Denmark	2023	Cohort study	C: 553 CG: 2677	SG, LAGB, GBP	6 months and 3 years	7
8	Singh P. et al. [23]	United Kingdom	2021	Retrospective matched controlled cohort study	C: 1126 CG: 2219	RYGB, SG, LAGB	3, 5 years	7
9	Madsen et al. [24]	Denmark	2019	Cross-sectional study	C: 96 CG: 48	RYGB	6 years	9
10	Miras, Alexander D. et al. [25]	United Kingdom	2019	Retrospective clinical trial	C: 82	RYGB, SG, LAGB	5 years	9
11	Richardson et al. [26]	United Kingdom	2018	Retrospective observational study	C: 32	RYGB	3 years	7
12	Chen Y. et al. [27]	United Kingdom	2017	Retrospective observational study	C: 102	Unspecified	4 years	8
13	Amin, Amin Mamoon et al. [28]	United Kingdom	2016	Retrospective cohort study	C: 152	Unspecified	3 years	8

VSG—vertical sleeve gastrectomy; gastric bypass surgery—GBP; Roux-en-Y gastric bypass—RYGB; laparoscopic sleeve gastrectomy—SG; laparoscopic adjustable gastric banding—LAGB; control group—CG; cases—C.

**Table 3:** Summary of studies investigating the short-term effects of bariatric surgery (BS) on diabetic retinopathy (DR) in patients with type 2 diabetes.

Study	Follow-Up	New-Onset DR	DR Worsening	DR Improvement	No Change in DR Status
Brynskov T. et al. (2016) [16]	1, 3, 6, and 12 months	6 months: 1/32 (3%) 12 months: 0/32 (0%)	12 months: 3/24 (13%) 5/24 (21%) at any follow-up visit	12 months: 4/24 (17%) 6/24 (25%) at any follow-up visit	12 months: 49/56 (87.5%)
Morén et al. (2018) [17]	1 year	12/73 (16%)	7/44 (16%)	8/44 (18%)	77/117 (66%)
Wenhuan Feng et al. (2019) [18]	1 year	DR at baseline vs. after 12 months S: 6/40 (15%) vs. 3/40 (8%) M: 7/36 (19%) vs. 7/36 (19%)			
Ozkan Sever et al. (2020) [19]	6 months and 1 year	Surgery: 17/21 (80.9%) had ocular complications Controls: 3/27 (11.1%) had ocular complications			
Åkerblom H. et al. (2021) [20]	1 year	S: 188/5321 (0.83%) C: 317/5321 (2.94%)	S: 42/5321 (0.79%) C: 45/5321 (0.85%)	No data	No data
Chandru S. et al. (2022) [21]	1 year	No data	1/8 (12.5%)	2/8 (25%)	7/8 (87.5%)
Anne S. Thykjaer et al. (2023) [22]	6 months	No data	S: 8/274 (2.9%) C: 34/404 (8.4%)	S: <5/11 (<45%) C: 15/76 (19.7%)	No data

DR, diabetic retinopathy; S, surgical; M, medical; C, controls.

**Table 4:** Summary of studies investigating the long-term effects of bariatric surgery on diabetic retinopathy in patients with type 2 diabetes.

Study	Follow-Up (years)	New-Onset DR	DR Worsening	DR Improvement	No Change in DR Status
Anne S. Thykjaer et al. (2023) [22]	3	Screening group (treated with BS): 13/249 (5.2%) Control group (BS not applied): 169/2131 (7.9%)		Screening group (treated with BS): 6/9 (66.7%)	No data
				Control group (BS not applied): 25/68 (36.8%)	
Singh P. et al. (2021) [23]	3.5	Screening group (treated with BS): 34/1069 (3.2%)	No data	No data	No data
		Control group (BS not applied): 96/2068 (4.6%)	Control group (BS not applied): 96/2068 (4.6%)	No data	
Madsen et al. (2019) [24]	6	No data	Screening group (treated with BS): 0/96 (0%)	Screening group (treated with BS): 50/96 (52%)	No data
			Control group (BS not applied): 46/48 (95%)	Control group (BS not applied): no data	
Miras, Alexander D. et al. (2019) [25]	5	0/24	6/24 (25%)	5/24 (20.8%)	13/24 (54.2%)
Richardson et al. (2018) [26]	3	9/47 (19.1%)	3/17 (17.6%)	11/17 (64.7%)	41/64 (64.1%)
Chen Y. et al. (2017) [27]	4	24/69 (34.8%)	2/33 (6.1%)	4/33 (12.1%)	72/102 (70.6%)
Amin, Amin Mamoon et al. (2016) [28]	3	29/106 (27.4%)	5/41 (12.2%)	5/41 (12.2%)	113/152 (74.3%)

DR, diabetic retinopathy; S, surgical; M, medical; C, controls.

## Data Availability

Not applicable.
